# Genome-wide DNA methylation in an animal model and human studies of schizophrenia: a protocol for a meta-analysis

**DOI:** 10.1136/bmjos-2021-100264

**Published:** 2022-08-25

**Authors:** Thabo Magwai, Fredrick Otieno Oginga, Bonginkosi Chiliza, Thabisile Mpofana, Khethelo Richman Xulu

**Affiliations:** 1 Department of Physiology, University of KwaZulu-Natal College of Health Sciences, Durban, South Africa; 2 Department of Chemical Pathology, University of KwaZulu-Natal College of Health Sciences, Durban, South Africa; 3 Department of Clinical Medicine, Kabarak University, Nakuru, Kenya; 4 Department of Psychiatry, University of KwaZulu-Natal Nelson R Mandela School of Medicine, Durban, South Africa; 5 School of Molecular and Cell Biology, Faculty of Science, University of the Witwatersrand, Johannesburg-Braamfontein, Gauteng, South Africa

**Keywords:** DNA, rats

## Abstract

**Introduction and objective:**

Neuropsychiatric disorders like schizophrenia are heterogeneous in that they occur because of the interaction of factors. These factors include but are not limited to genetic, epigenetic, neurobiological and environmental factors. Methylation of DNA, like other erpigenetic modifications, is risk factors for neuropsychiatric disorders. Candidate gene approach projects have produced contradictory results to find candidate gene methylation. The current genome-wide studies have limitations.

**Search strategy:**

An exhaustive search strategy was designed to recover studies on genome-wide DNA methylation in schizophrenia patients or schizophrenia rat models. The Medline (PubMed), SCOPUS and Web of Science, databases were searched, giving 4077 references in total.

**Screening and annotation:**

Studies will undergo two phases of screening, title and abstract screening and article screening, for inclusion by two reviewers. A third reviewer will resolve any disagreements in the article screening phase. Data will be collected using the Systematic Review Facility (http://syrf.org.uk/) tool. All included studies will undergo study quality and risk of bias assessment.

**Data management and reporting:**

Data will be extracted and used to calculate effect sizes. For the purpose of this meta-analysis, a random effects model will be used to combine effect sizes. Heterogeneity will be assessed, and the sources identified. A risk-of-bias assessment will be carried out to assess the quality of the studies. An assessment of publication bias will also be carried out.

**Ethics and dissemination:**

No ethical approval is required as there are no participants in the study. We will follow the Preferred Reporting Items for Systematic Reviews and Meta-Analyses reporting guidelines and disseminate the findings through publication and conference presentation

**PROSPERO registration number:**

CRD42021283159.

## Introduction

Schizophrenia is defined as a mental illness whose attributes include disconnection of thoughts, ideas, identity and emotions.^1–5^ The symptoms of the disease, both positive and negative, are due to dysregulation of the neural pathways.^2 3 5 6^ Despite the increased research in the field schizophrenia, there remains still a gap in knowledge related to the pathophysiology, biomarkers for the prediction of the onset of schizophrenia.^5 7–10^ The heritability schizophrenia has been approximated to range from 79% to 81%.^11–13^ It has been hypothesised that this may be because schizophrenia genetic risk loci are found in the introns and promoter gene regions, leading to a suspicion that the regulation of genes may be of importance in the development of the disease.^14^ The risk loci are thus thought to be linked to the expression of genes,^15–17^ thus suggesting that epigenetics is an arbitrator of genetic risk in the development of schizophrenia.^5 14^

DNA methylation is the most documented epigenetic marker.^18 19^ DNA methylation is evident by the attachment of a methyl group at the cytosine-guanine dyads (CpG) dinucleotide.^5 20 21^ Initially, DNA methylation projects interrogated alterations in candidate genes like *RELN*,^22 23^ *COMT*^24^ and *GAD67*.^25^ Due to conflicting results with candidate genes, researchers opted for genome-wide DNA methylation methods for a more comprehensive whole-genome coverage than methylation of a targeted gene.^14 26^ Even with the growing number of genome-wide methylation studies, there remains still a problem with the pinpointing consistent schizophrenia-specific DNA methylation patterns. This challenge is due to the lack of repeatability of results in genome-wide studies. This lack of reproducibility has been thought to be due various limitations of studies in current literature.^5^ The current limitations are the small sample size, the type of samples used for analysis,^27^ medication use,^28^ smoking,^29^ tissue and cell-type heterogeneity^30^ and epigenetic methods used.^31^ One way to mitigate the effects of these limitations is to perform a meta-analysis, which will provide a clear picture of previously conducted studies and provide future directions for further investigations.

## Methods

### Research question and search strategy

This review aims to assess and summarise the findings of studies that characterised the genome-wide DNA methylation in patients or rodent models with schizophrenia compared with controls. The study will also explore the possibility of blood-based DNA methylation as a biomarker for schizophrenia diagnosis, prognosis or therapeutic marker.

### Objectives

1. To determine differentially methylated regions (DMR) or positions (DMP) in humans or rodents models of schizophrenia compared with those without schizophrenia.
2. To determine the common DMR or DMP related to schizophrenia, controlled for common cofounders.
3. To determine DMRs or DMPs related to schizophrenia that is common between brain tissue and peripheral tissue.

### Searches

The MEDLINE (PubMed), Web of Science and Scopus databases will be used for the electronic search of published studies. The searches will be from inception to 30 September 2021. The search will be limited only to publications in the English language and any publication that can be converted to English. Keywords were chosen based on the combinations of terms for schizophrenia and genome-wide DNA methylation. The search strategy used is listed in table 1. A manual search of the reference lists of included studies and relevant reviews related to the study question will be conducted to identify additional articles for inclusion.

**Table 1:** Different syntax used to search

Database	The syntax used to search
MEDLINE (PubMed)	(((Schizophrenia) OR (Psychosis) OR (Mental disorder) OR (Mental illness)) AND ((genome wide DNA methylation) OR (genome-wide DNA methylation) OR (epigenome wide DNA methylation) OR (epigenome-wide DNA methylation) OR (genome wide methylation) OR (genome-wide methylation) OR (epigenome wide methylation) OR (epigenome-wide methylation) OR (whole genome DNA methylation) OR (whole-genome methylation)))
Scopus and Web of science	(((schizophrenia) OR (psychosis) OR (mental AND disorder) OR (mental AND illness)) AND ((genome AND wide AND dna AND methylation) OR (genome-wide AND dna AND methylation) OR (epigenome AND wide AND dna AND methylation) OR (epigenome-wide AND dna AND methylation) OR (genome AND wide AND methylation) OR (genome-wide AND methylation) OR (epigenome AND wide AND methylation) OR (epigenome-wide AND methylation) OR (whole AND genome AND dna AND methylation) OR (whole-genome AND methylation)))

### Study selection and inclusion/exclusion criteria

All original research articles, with no restrictions on the date, will be included, which reported genome-wide DNA methylation in schizophrenia patients or schizophrenia rat models. Studies using brain tissue and peripheral tissue (blood) will be included. Rodent studies that used schizophrenia treatment will be included in the meta-analysis. The meta-analysis will consist of male and female participants in human and rodent studies. Only animal models using rodents will be included in the meta-analysis. Exclusion criteria to be used are as follows: studies written in other languages either than English and cannot be translated to English, articles that have no full text available (even after an attempts to acquire the full text from the author), studies with no new data like review articles, systematic reviews, book chapters and conference abstracts, and studies that used targeted gene approaches and not genome wide approach. The review will also exclude case studies, cross-over studies, studies with no separation of control group, in vitro studies, ex vivo studies and studies using in silico models for schizophrenia investigation.

The selection of studies will include two screening phases, the title and abstract screening phase and the article screening phase. For the title and abstract screening phase, studies obtained from the database searches will undergo title and abstract screening by two independent researchers. Any study selected by at least one reviewer will go through full-text screening. The full text will be obtained from all the studies that passed the title and abstract screening for the article screening phase. Two researchers will independently assess the full text for eligibility. The third reviewer will resolve any discrepancies between the two reviewers.

### Collection of study characteristics

The Systematic Review and Meta-analysis Facility (SyRF) platform (http://syrf.org.uk/) will be used to the collection of qualitative and quantitative data from the included studies. Studies will be imported to the SyRF platform (app.syrf.org.uk) for annotation and extraction of data. Annotations include characteristics of (1) animals or patients (species, strain, age, sex, age at diagnosis (humans), tobacco smoking, cause of death, alcohol use (humans)), (2) control and schizophrenia genome-wide DNA methylation, (3) medication use (human studies) or schizophrenia induction (animal studies) (name, dose, mode of administration, duration of administration), (4) of experimental designs (genome-wide DNA methylation used, CpG and/or non-CpG coverage, sample size, tissue and/or cell type used for analysis), (5) cognitive outcomes measured or symptoms of patients (where possible) (like behaviour task performed, timing of outcome assessment relative to diet/intervention administration, symptoms for humans) and (6) additional (eg, transcriptomics data, other reported omics) outcomes reported.

### Study quality appraisal and risk of bias

Studies will be assessed for selection bias, performance bias, detection bias, attrition bias (for animal studies), reporting bias and other biases. Using the CAMARADES checklist for study quality, adapted as follows: The Downs and Black checklist will be used for the assessmenmt of quality and preferences.^32^ The checklist comprises four domains, namely, the reporting of bias, external validity, internal validity and selection bias. The scores are categorised as excellent (score between 26 and 27), good (score between 20 and 25), fair (score between 15 and 19) and poor (score between 0 and 14).^33^ Additionally, a modified version of the Systematic Review Centre for Laboratory animal Experimentation will be used to assess risk bias for animal studies.

### Extraction of outcome data

For animal studies, outcome data will be collected for behavioural tests, including an examination of executive function, learning, and spatial and non-spatial memory. Outcome data will be collected for mental health diagnosis studies using human participants. For each study, the significant DMR’s or DMP’s with an false discovery rate (FDR) cut of of ≤0.05, genomic position of DMR’s, FDR, affected genes and correlation value will be collected. Additionally, sample size, species origin of samples used, cell or tissue type used, the method used for DNA methylation assessment will be extracted. Should there not be any issues with results from different methods, the results will be controlled for themethod used statistically or by study selection. For studies using human samples, different outcomes like age of diagnosis, medication use, tobacco smoking, other diagnoses for patients with multiple mental disorders diagnosis and use of drugs will be collected. For animal’s studies, outcomes like the method of inducing schizophrenia, results of behavioural testing and species of animal used will be reported. The type of medication used for both rodents and human studies where medication/treatment was used will be collected. Additionally, data on covariates, such as differentially expressed genes (DEG’s), microRNA, proteomics and metabolomics, will also be collected where they are reported.

Quantitative data will be extracted from the study using either a digital ruler tool or the embedded Graph2Data tool, if available. Missing values will not be imputed. Authors will be contacted for further information if all data cannot be ascertained. The authors of studies will be reached where inadequate data are provided to acquire enough information. Extracted data will be managed using Review Manager.

### Quantitative analysis

STATA V.17.0 software will be used for statistical analysis. Pooled risk ratios (RRs) will be determined and reported with a 95% CI. For dichotomous data, an RR with a 95% CI will be measured. The RR has been shown to be more instinctive than OR. For continuous data, a weighted means differences (with 95% CI) will be used. Standardised mean differences (95% CI) will be used in the event thatof different measures. For skewed data and qualitative data, adescriptive presentation will be used.

### Heterogeneity

The heterogeneity will be quantified by using χ^2^ test and I^2^ metric. A random effect model priori will be used in the study. A random effect model has been chosen as the conclusion will be generalised beyond the included studies. Any decrease in heterogeneity will be determined using subgroup analysis for categorical data and meta-regression analysis for continuous data. A narrative synthesis will be presented for any variables with insufficiently homogeneity or if the data are insufficient for meta-analysis.

### Analysis of subgroups or subsets

Depending on the number of studies, a subgroup analysis will be performed with a minimum of two studies for a meta-analysis. Suppose an adequate number of studies for a meta-analysis, subgroup analysis will be performed.

The following subgroups will be investigated if possible:

1. Human postmortem brain tissue compared with those without schizophrenia.
2. Human peripheral tissue (blood) compared with those without schizophrenia.
3. Animal brain tissue compared with those without schizophrenia.
4. Animal peripheral tissue (blood) compared with those without schizophrenia.
5. Common DMR’s or DMP’s in brain tissue and peripheral tissue for animals and humans.
6. Common traits like common medications and tobacco use compared controls.

An exploratory analysis will be performed to estimate how moderators of interest to the literature may influence results. We will explore the relationship between factors like age, use of medication, smoking and symptom severity, results of the behavioural test (animal studies) on genome-wide methylation differences between participants with schizophrenia and comparison groups. The effects of moderators will be assessed by Bayesian hierarchical models (meta-regression).

### Sensitivity and publication bias

Publication bias will be tested by using the funnel plot analyses, and the Egger method will be used to quantify publication bias.^34^ The trim-and-fill methods will be used to determine the impact of publication bias where there is publication bias.

## Discussion

Preclinical systematic reviews and meta-analyses are valuable tools in combining large numbers of projects’ data with conflicting results as seen in genome-wide DNA methylation studies. These reviews can address limitations and help guide the design and content of future preclinical research and human clinical studies.^35^ Genome-wide DNA methylation studies suffer from complications with the reproduction of results. This challenge is due to sample size, type of sample used,^27^ medication use,^28^ tobacco smoking,^29^ tissue and cell heterogeneity,^30^ and DNA methylation methods used.^31^ Also, these studies have used different samples from human schizophrenia patients and various animal models for schizophrenia. Other studies have also captured additional data like transcriptomic data, medication use, further diagnosis, sample and tissue.

### Strengths and limitations

The study’s strength is that it will review data from both human participants and rodents with schizophrenia. The study intends to conduct a meta-regression in both groups and compare the outcomes of the two groups. Furthermore, with our subgroup analysis, we could gather information on potential biomarkers for schizophrenia. The review also has some potential limitations. The first limitation is that the included studies might have high variability in their experimental designs and conduct, which may influence and report the analyses. The second limitation is that the summary effect size may be overestimated due to publication bias. However, this could be addressed by using statistical methods to calculate a true estimate of the effect, filtering out the influence of publication bias.

## Data Availability

Data sharing not applicable as no datasets generated and/or analysed for this study. No data are available. All data relevant to the study are included in the article. All data relevent to the study are included in the article.
